# Prioritizing and Analyzing the Role of Climate and Urban Parameters in the Confirmed Cases of COVID-19 Based on Artificial Intelligence Applications

**DOI:** 10.3390/ijerph17103730

**Published:** 2020-05-25

**Authors:** Sina Shaffiee Haghshenas, Behrouz Pirouz, Sami Shaffiee Haghshenas, Behzad Pirouz, Patrizia Piro, Kyoung-Sae Na, Seo-Eun Cho, Zong Woo Geem

**Affiliations:** 1 Department of Civil Engineering, University of Calabria, 87036 Rende, Italy; S.shaffiee@yahoo.com (S.S.H.); Sami.shaffiee@gmail.com (S.S.H.); patrizia.piro@unical.it (P.P.); 2 Department of Mechanical, Energy and Management Engineering, University of Calabria, 87036 Rende, Italy; behrouz.pirouz@unical.it; 3 Department of Computer Engineering, Modelling, Electronics and Systems Engineering, University of Calabria, 87036 Rende, Italy; behzadpirouz@gmail.com; 4 Gil Medical Center, Gachon University, Incheon 21565, Korea; ksna13@gmail.com (K.-S.N.); arztin01@hanmail.net (S.-E.C.); 5 College of IT Convergence, Gachon University, Seongnam 13120, Korea

**Keywords:** sustainable development, COVID-19, artificial intelligence, PSO, DE, feature selection

## Abstract

Nowadays, an infectious disease outbreak is considered one of the most destructive effects in the sustainable development process. The outbreak of new coronavirus (COVID-19) as an infectious disease showed that it has undesirable social, environmental, and economic impacts, and leads to serious challenges and threats. Additionally, investigating the prioritization parameters is of vital importance to reducing the negative impacts of this global crisis. Hence, the main aim of this study is to prioritize and analyze the role of certain environmental parameters. For this purpose, four cities in Italy were selected as a case study and some notable climate parameters—such as daily average temperature, relative humidity, wind speed—and an urban parameter, population density, were considered as input data set, with confirmed cases of COVID-19 being the output dataset. In this paper, two artificial intelligence techniques, including an artificial neural network (ANN) based on particle swarm optimization (PSO) algorithm and differential evolution (DE) algorithm, were used for prioritizing climate and urban parameters. The analysis is based on the feature selection process and then the obtained results from the proposed models compared to select the best one. Finally, the difference in cost function was about 0.0001 between the performances of the two models, hence, the two methods were not different in cost function, however, ANN-PSO was found to be better, because it reached to the desired precision level in lesser iterations than ANN-DE. In addition, the priority of two variables, urban parameter, and relative humidity, were the highest to predict the confirmed cases of COVID-19.

## 1. Introduction

Sustainable development is an approach planned to improve human life and considers the development process while simultaneously analyzing related impacts [1,2,3]. Its critical role is becoming more and more important every day. After great efforts, in 2015, United Nations member states approved the 2030 Agenda for Sustainable Development, which includes 17 Sustainable Development Goals (SDGs) [4].

The main aspects of sustainable development are simultaneous consideration of environmental, social, and economic, and if the policies of governments consider these three factors separately, the effect on sustainable development can be negative [5,6]. There are many evaluation methods to analyze the previous efforts on sustainable development [7,8,9], and the selected techniques depend on the study goals [10,11,12,13,14]. 

Although sustainable development is not a new concept, these methods neglect important elements. Among them, epidemic diseases can have a temporary or permanent negative impact [15,16]. Moreover, urbanization also plays an important role in this regard, since one of its impacts is the increase of the population density, which can affect the epidemic rate of diseases directly [17,18,19,20]. COVID-19 is a recent pandemic disease [21]. There are several studies about coronavirus spread, the time the virus remains in the environment, the epidemic rate, and the mathematical models for the prediction of COVID-19 contagions [22,23,24,25,26]. 

Chen et al. developed a time-dependent mathematical model for the prediction of the total number of confirmed cases [27]. Pirouz et al. used artificial intelligence (AI) algorithm to study the correlation between environmental parameters and COVID-19. They found a significant correlation between urban and climate parameters and the number of confirmed cases of COVID-19 [28]. Hu et al. developed a predictive model for the transmission period of the COVID-19 using AI techniques. Their results showed a high-performance of AI in predicting the outbreak of coronavirus [29]. 

Kampf et al. investigated the time the coronaviruses survive on different surfaces. Their results show that COVID-19 can survive up to nine days, depending on the environment temperature and materials of the surface [30]. Grant and Giovannucci carried out a study about the impact of temperature on COVID-19 and showed that most patients had been exposed to temperatures between 3 and 17 degrees Celsius, and that the infection rates are lower in tropical regions [31]. In a previous study, Chan et al. had analyzed the effects of temperature and humidity on another type of coronavirus, SARS. According to this study, when the temperature increases, the survival time of the virus on the surfaces can decrease, and this leads to a lower epidemic rate [32].

The review of these previous studies and the size of the pandemic reveals that new coronavirus (COVID-19) as an infectious disease has undesirable social, environmental, and economic impacts and might lead to serious challenges and threats in many societies. Therefore, the paper will prioritize and analyze the role of certain environmental parameters, including daily average temperature, relative humidity, wind speed, and an urban parameter, population density, which have essential roles in reducing the negative impacts of this global crisis by using artificial intelligence techniques.

## 2. Methodology

Two analytical approaches have been used. At first, by using a multivariate linear regression (MLR) model, the correlations between the three climate parameters, including average temperature, humidity, and wind speed, and the confirmed cases of COVID-19 were investigated, and the required datasets for artificial intelligence prepared. Then, two artificial intelligence techniques based on ANN, including the PSO algorithm and DE algorithm, have been used for predicting the confirmed cases of COVID-19 and to prioritize and reduce the input parameters. 

Analysis Conditions:

- The analysis factors are the population density of each region, average daily temperature, relative humidity, wind speed, and the positive cases in the following days;
- Since the incubation period of the virus is about 14 days, the sum of previous positive cases up to 14 days previously has been considered;
- The analysis period is from 14 February 2020 to 24 March 2020.

In addition, it must be noticed that there are some delays between the exact dates when patients got infected by the COVID-19, and the dates when confirmed cases were registered in the media as follows:

- The incubation period of COVID-19 varies from about 2 to 14 days [33];
- The lab tests of COVID-19 were on patients with symptoms [34];
- The symptoms of COVID-19 occur after 3 to 5 days [35];
- The results of the laboratory tests took one day to be ready [36,37];
- The daily announcement of new confirmed cases of COVID-19 usually refers to one day before [38].

Therefore, to find an appropriate correlation between weather data and confirmed cases, the climate factors have been shifted backward from one to nine days with respect to observations, and by the MLR method, the best correlation for each region has been selected. The results are presented in Appendix A, and have been used as a database in the artificial intelligence method.

It is evident that using daily positive cases—and especially data from one day before—could not be correct, due to the incubation period of COVID-19 (2 to 14 days), the symptoms of COVID-19 (which occur after 3 to 5 days), and finally, the fact that the lab tests of COVID-19 were on patients with symptoms. Therefore, the new positive case in date X will depend on the accumulative positive cases up to 14 days ago (Date X-14). This variable cannot reach a plateau since it represents the accumulation of 14 days, not the period from start to end, as presented in Appendix B. 

### 2.1. Artificial Intelligence Methods

#### 2.1.1. Artificial Neural Network (ANN)

**Figure 1:**
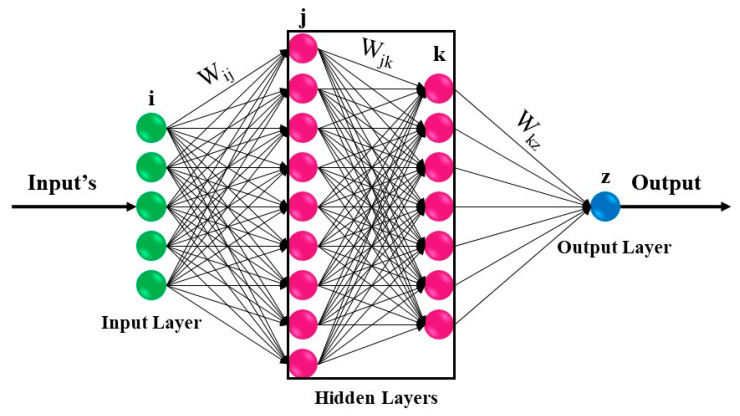
The basic form of multilayer perceptron artificial neural network (ANN) [61].

The human brain, as a complex natural system, is unique in its kind. Some of the processes in this natural system are so complex that their processing is also complex for many super systems [39,40,41,42,43,44]. Analytical processes are very complex, because of the high speed and power of information processing by brain cells. Researchers were enabled to design advanced methods for solving various problems of real world inspired by the function of the human brain. Hence, artificial intelligence (AI) is considered one of the most successful achievements of computer science, simulating the behavior of the human brain in data analysis [45,46,47,48,49,50,51]. One of the AI branches is the artificial neural network (ANN). This information processing system, by a simulating strategy like communication between brain neurons, has become a tool for analyzing complex and real systems. In recent years, ANN models have been developed to overcome the difficulties presented by health issues [52,53,54]. Many types of computational models have been introduced as general neural networks. The multilayer perceptron (MLP) model is one of the most efficient ones, and has been used in a variety of activities. The MLP is a supervised artificial neural network with at least three layers, including the input layer, hidden layer, and output layer. The basic form of an artificial neural network includes a set of connected units or nodes (artificial neurons), and connections (weights). The connections can transmit a signal from one neuron to another, as shown in Figure 1. Depending on a particular problem, the number of neurons and the hidden layer can be changed to find the best prediction model [55,56,57,58,59,60]. The performance indicators of the algorithm evaluate the difference between the predicted values and the last layer (output). The process of training and evaluating the results in this algorithm continues until a desirable convergence is reached, and then it stops.

#### 2.1.2. Particle Swarm Optimization (PSO) Algorithm

**Figure 2:**
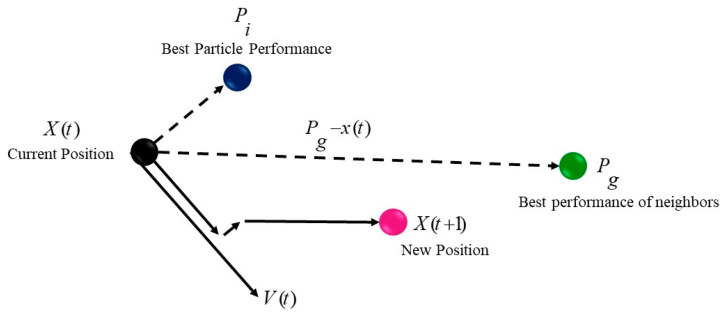
The update of the velocity and position vectors [61].

In recent years, the use of artificial intelligence by many researchers to solve complex and uncertain problems has become widespread [62,63,64,65,66,67,68,69,70,71], and there have been especially successful applications in the health problems [72,73,74,75,76]. One of these advanced techniques is the particle swarm optimization (PSO) algorithm, first introduced by Kennedy and Eberhart [77,78,79]. The algorithm was designed to simulate the swarm behavior of particles and to inspire the movement of birds and flocks. The PSO algorithm has been used successfully for modeling in engineering and academic applications. In this algorithm, each particle in the particle set is considered as a potential solution that the process of this algorithm begins with the generation of a random particle set. Then, the process continues by moving the set of particles to search for an optimal answer in the search space. In addition, if there is a D-dimensional set, including N particles, each *i* particle in this set is indicated with an *X_i_* vector that includes vectors of position and velocity. In fact, the PSO algorithm differs from other algorithms in having a velocity vector. The new velocity vector and the new position vector of each particle are updated based upon Equations (1) and (2) in each moment. They depend on the particle’s best position (Pbest) and the global best position (Gbest) [80].

(1) $$V_{i}^{\left(k+1\right)}=wV_{i}^{k}+c_{1}r_{1}.(pbest_{i}-X_{i}^{k})+c_{2}r_{2}.(gbest-X_{i}^{k})$$

(2) $$X_{i}^{\left(k+1\right)}=X_{i}^{k}+V_{i}^{k}$$

where $X_{i}^{k}$ and $V_{i}^{k}$ are the current position and velocity of the particle *i*, respectively, and $V_{i}^{\left(k+1\right)}$ and $X_{i}^{\left(k+1\right)}$ its new position and velocity. The parameter w is called the inertia weight, and varies between 0.4 and 0.9. The $r_{1}$ and $r_{2}$ are two random numbers within [0, 1]. The constants *C*_1_ and *C*_2_, called the individual learning factor and social learning factor, are positive and must satisfy Equation (3). Figure 2 shows the update of the velocity and position vectors of a particle in the set [81,82].

(3) $$c_{1}+c_{2}\leq 4$$

**Figure 3:**
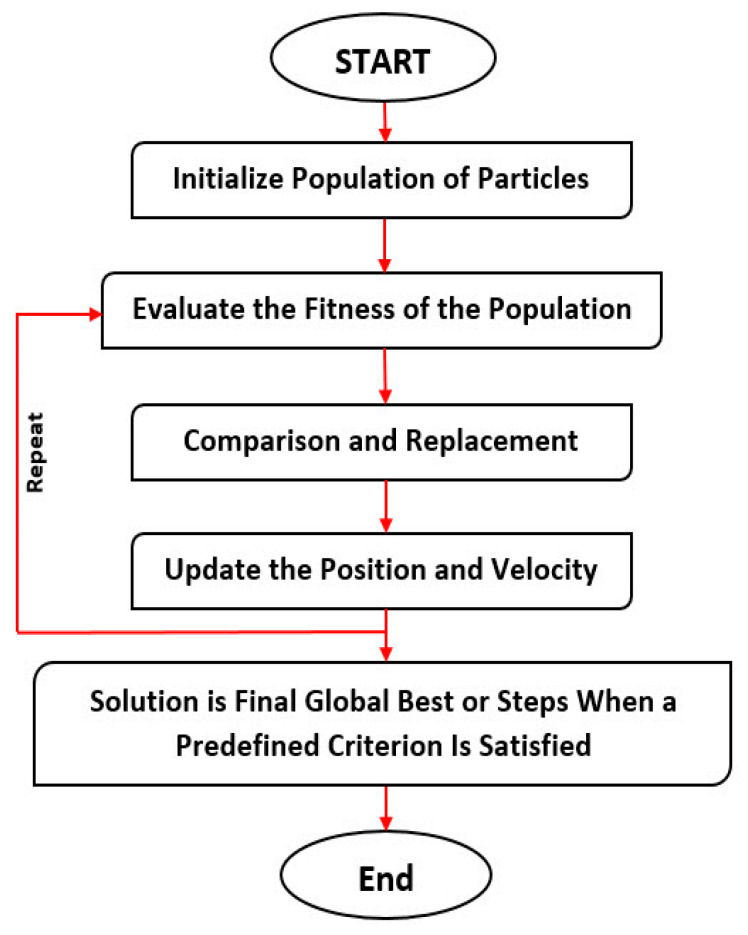
The particle swarm optimization (PSO) algorithm flowchart [83].

Eventually, all particles converge to the optimal point after a thorough search. Figure 3 presents the flowchart of the PSO algorithm.

#### 2.1.3. Differential Evolution (DE) Algorithm

**Figure 4:**
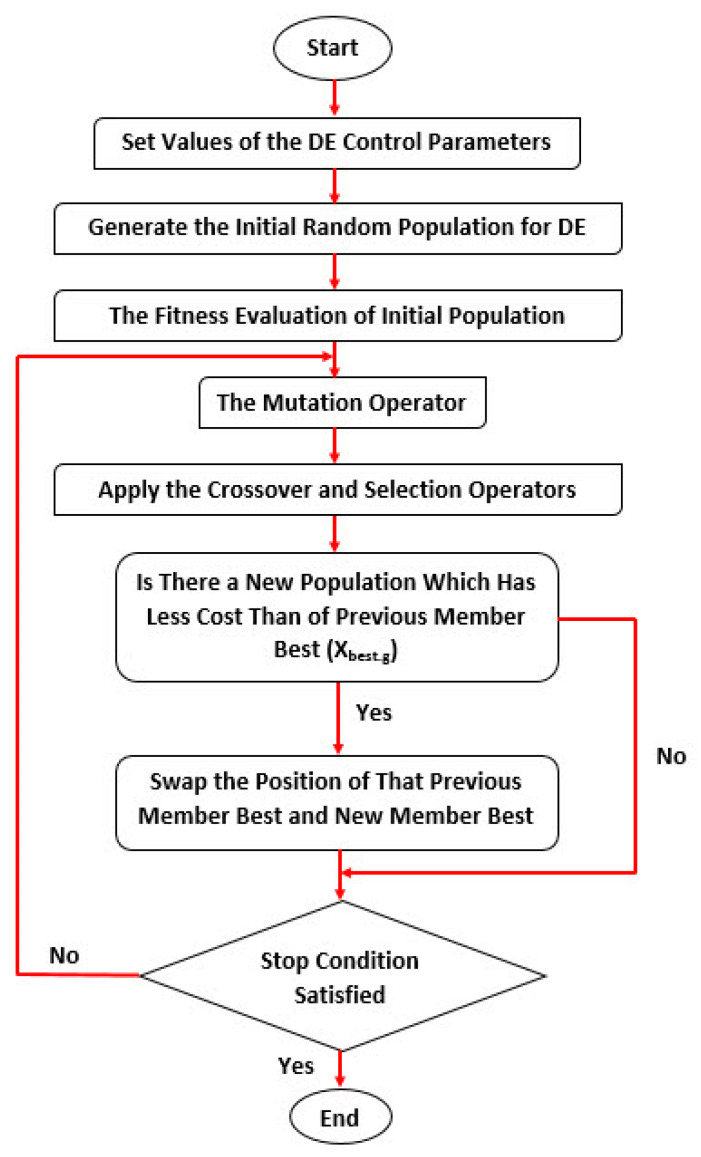
The differential evolution (DE) algorithm flowchart [91].

The differential evolution (DE) is an evolutionary computation that is suitable for dealing with complex problems in the real world. The DE algorithm is a population-based algorithm that was proposed by Price and Storn for solving the continuous value problems [84,85,86,87]. Then, in the following years, the method developed and used for solving binary and discrete problems. The DE algorithm has been widely applied as an optimization algorithm to solve complex problems in various engineering sectors. The DE algorithm and some Meta heuristic algorithms like genetic algorithms have similar operators, including crossover, mutation, and selection. However, there are some differences among them, like the lack of local search in genetic algorithm, while the DE algorithm supports local search. In addition, the DE relies on mutation operation while the genetic algorithm relies on a crossover. Like other evolutionary algorithms, the DE starts by randomly generating the initial population. Then, after initialization, the search space is expanded by the mutation. The $V_{i}^{g}$ is the mutant solution vector of $X_{i}^{g}$ which is calculated based on Equation (4) [88].

(4) $$V_{i}^{g}=X_{r1}^{g}+F^{k}.(X_{r3}^{g}-X_{r2}^{g})$$

where $F^{k}$ is the scaling factor varying in the range [0, 1] and determines the length of the mutation step. $X_{r1}^{g},X_{r2}^{g}\;and\;X_{r3}^{g}$ are solution vectors that are randomly selected, with the condition expressed by Equation (5) [89].

(5) $$\begin{array}{l} X_{r1}^{g},X_{r2}^{g},X_{r3}^{g}|r_{1}\neq r_{2}\neq r_{3}\neq i\\ i=[1,2,3,\ldots ,NP] \end{array}$$

where “*i*” is the index of the current solution. The trial vector ($U_{ij}^{g}$) is produced by mixing the mutated vector and the parent vector in a crossover operation based on Equation (6) [90].

(6) $$U_{ij}^{g}=\left\{ \begin{array}{l} V_{ij}^{g}\;\;\;\;Rand_{j}\leq CR,\\ X_{ij}^{g}\;\;\;\;Rand_{j}>CR, \end{array}\;\;\right.j=1,2,3,....,n$$

where *Rand_j_* is a randomly chosen real number in the interval between 0 and 1. The CR is a crossover constant. If the *Rand_j_* is less than or equal to *CR*, the trial vector ($U_{ij}^{g}$) is inherited from the mutant solution vector, otherwise, the CR is considered equal to $X_{ij}^{g}$. The flowchart of the DE algorithm is shown in Figure 4.

### 2.2. Subsection

**Figure 5:**
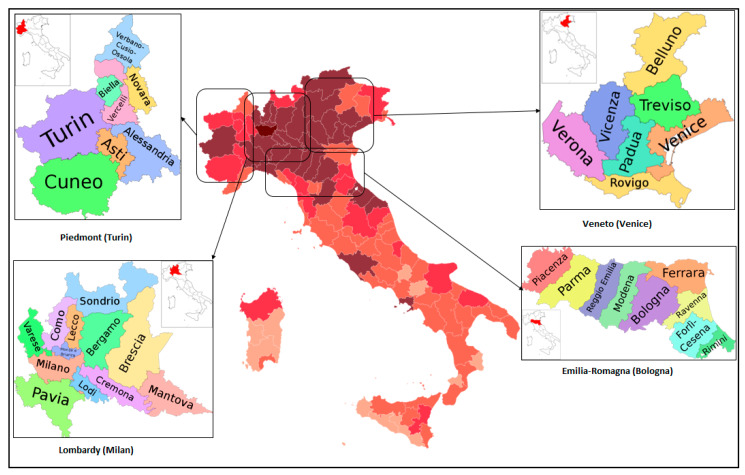
Locations of the case study regions, Italy [94,95,96,97,98].

In this research, the case studies are the four regions in Italy with the largest numbers of confirmed cases of COVID-19, namely Lombardy (Milan), Piedmont (Turin), Veneto (Venice), and Emilia-Romagna (Bolonia), whose general data are presented in Table 1. The locations of the case studies are shown in Figure 5.

## 3. Model Development

### 3.1. PSO Modelling

**Figure 6:**
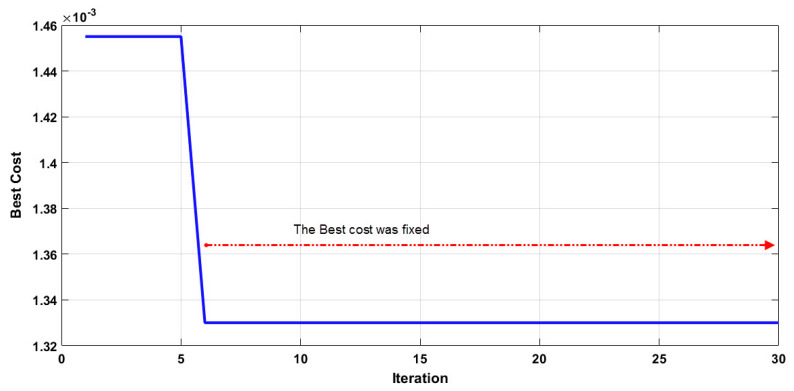
The best cost per each iteration by PSO algorithm.

The main goal of PSO is to train the artificial neural network for determining the feature selection of confirmed cases of COVID-19, and the reduction of them under the highest relationship between several independent variables and the dependent variable. For this purpose, three notable climate parameters, namely daily average temperature, relative humidity, and wind speed, and one urban parameter (population density × positive cases up to 14 days before), were considered as input data set, and confirmed cases of COVID-19 were considered as the output dataset. It is worth mentioning that the 4 input parameters are evaluated and reduced to 2. Firstly, before modeling, the control parameters of an algorithm should be selected. There are no specific rules, and most of them are considered based on the experts’ opinions and previous studies [61,82]. Hence, a number of different modeling are done to determine an appropriate value for control factors, for instance, the size of a hidden layer of ANN was selected for 10, 20, and 30, the maximum iteration value was considered as 15, 20, 25, 30, 40, and 50 and the swarm sizes as 5, 10, 20, 30, and 40. Secondly, after the initial analysis and trial and error, the best developed model was constructed with a structure shown in Table 2. Finally, the developed model was implemented for determining the best answer with 2 parameters. The obtained result of the best cost in each iteration is shown in Figure 6 for 2 parameters, respectively. In fact, the best cost in each iteration shows the performance function of the algorithm depends on the values of error in each iteration of modelling. It should be noted that we consider the mean squared error (MSE) for evaluation of the performance, and 70% of data set were considered for training, and the rest were considered for validation (15%) and testing (15%) [99].

According to Figure 6, it is evident that after the sixth iteration with 0.00133, the best cost was reached, and the model achieves a worthy convergence, and it was fixed to the end of the iteration. In addition, the model reduced the number of parameters from 4 to 2 that, in fact, reveal that the urban parameter and relative humidity were the priority of the model.

### 3.2. DE Modelling

**Figure 7:**
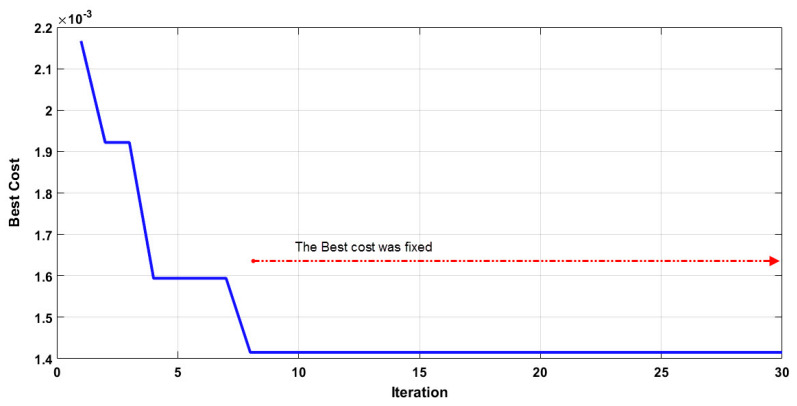
The best cost per each iteration by DE algorithm.

As mentioned earlier, the DE algorithm is used for training the artificial neural network to apply the feature selection with the four climate parameters, namely daily average temperature, relative humidity, and wind speed, and one urban parameter (population density × positive cases up to 14 days before) considered as the input data set, and the confirmed cases to COVID-19 considered as an output dataset. At first, the control parameters of DE algorithm are determined to find the optimum weights and biases of ANN model that can converge faster and accurately. For this purpose, similar to PSO model, the crossover probability coefficient was selected as 0.2, and other parameters were determined by trial and error method from previous studies and experts’ opinions [87,88]. In addition, the datasets for modeling were randomly divided into several subsets, including 70% for training and the rest for validation (15%) and testing (15%) [99]. Hence, population sizes of algorithm of 5, 10, 20, 30, and 40 were selected, and the maximum iteration was used with a range of values equal to 15, 20, 25, 30, 40, and 50. The values of 10, 20, and 30 were chosen for the size of the hidden layers of ANN. After the initial evaluation, the optimized model selected with the values of 5, 15, and 30 for the hidden layer, population size, and the maximum number of iterations, respectively. The process of optimization based on iterations is presented in Figure 7, which shows that the process reached the desired precision level of best cost with the value of 0.0014 from the 8th iteration, and it was fixed from the 8th to the 30th iteration.

The developed model by DE algorithm determined the urban parameter and relative humidity as priorities of prediction in this research. More discussions regarding the comparison of algorithms’ performances and the priorities of the parameters in the forecast will be given in the following section.

## 4. Discussion

In this research, two machine learning techniques of artificial intelligence, namely ANN based on the PSO algorithm and DE algorithm, were used for prioritizing climate and an urban parameter based on the feature selection process. Both developed models based on PSO and DE algorithms selected the urban parameters and relative humidity in the feature selection process, and the reduction of number of parameters. In fact, at first, these models calculated and achieved the best relationships between the output and all inputs based on the values of best cost, then the models considered the features as a binary choice, and finally they could find out that the best values of best cost with these two features are very close to the values of the best cost of all features. The developed model by the PSO algorithm achieved a suitable convergence with good accuracy in the sixth iteration, while the developed model by DE algorithm reached an appropriate convergence in the eighth iteration. Consequently, it is clearly seen that, although there is no salient difference between the performances of the two models, the model developed by PSO algorithm has a better performance in this specific problem, based on the best cost value and the rate of convergence.

Our results are in good agreement with those of Chan et al. [32] about the important role of humidity in another type of coronavirus, SARS, and of Pirouz et al. [28], that identified relative humidity as the higher-impact weather parameter. 

For further evaluation, the obtained results were validated by multivariate linear regression (MLR) technique and partial least squares regression (PLSR). For this, since for all four case studies, the correlations can be based on the two variables of humidity and urban parameter, the simplified final MLR and PLSR models are as follows:

- Prediction of   MLR    y = 169.96 + 0.000284 X 1 + 0.59 X 2,     R2 = 0.76
- Prediction of  PLSR   y = 193.26 + 0.00028 X 1 + 0.257 X 2,     R2 = 0.76

where X1 is the urban parameter, and X2 is the relative humidity. Therefore, the analysis shows that the prediction of confirmed cases of COVID-19 could be made by using two factors of relative humidity and urban parameter (population density X positive cases up to 14 days before).

**Figure 8:**
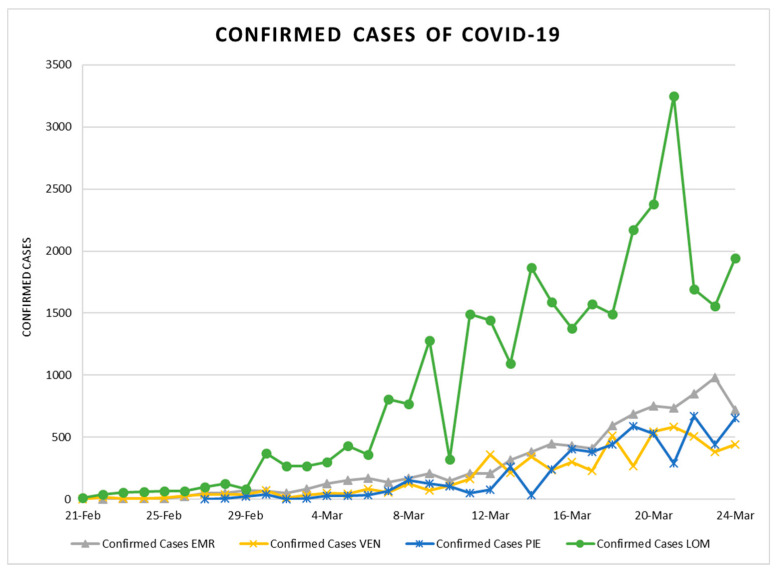
Daily confirmed cases of COVID-19 in four regions.

**Figure 9:**
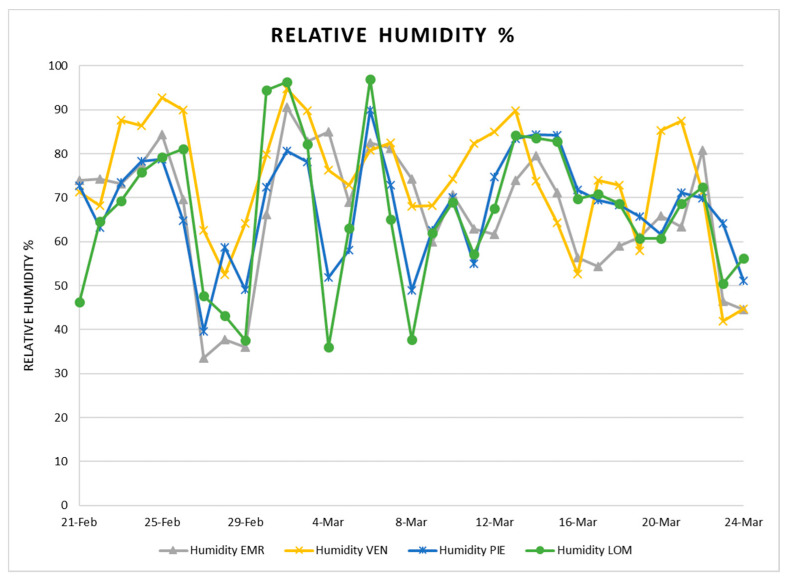
Relative humidity in four regions.

The trend of confirmed cases in four regions is shown in Figure 8, and the daily relative humidity in Figure 9. According to Figure 8, it is evident that the number of infections in all regions were equal at the beginning, but in Lombardy with the highest density increased more. Analysis of relative humidity exhibits that the fluctuations of humidity percentage was the highest in Lombardy, and then in Piedmont, as well as the number of confirmed cases that in both case studies show daily fluctuations. 

In addition, the analysis determined that even in one climate type, as the climate type of all four regions is humid subtropical, there might be other essential variables such as population density that affect the final results. In addition, the differences in the fluctuation of relative humidity in one type of climate as an influential parameter in the number of confirmed cases of COVID-19 show that for other types of climates, the selection of different case studies is a necessity.

Finally, it is worth mentioning that the results of this research are derived explicitly for the studied regions in the north of Italy with a humid subtropical climate, and they should not be used directly in other countries. For possible future work referring to other countries, it is recommended to see the effectiveness of the other parameters, such as different climate conditions and urban parameters. In addition, the outdoor humidity could affect the indoor humidity, which might be another important parameter for future analysis. In addition, it might be worth studying whether the use of other machine learning methods may improve our results.

## 5. Conclusions

With regard to the immense importance of sustainable development to improve the conditions of today’s and future generations, evaluating its challenges and obstacles has considerable effects on government decisions. Hence, in this research, the pandemic novel coronavirus infection (COVID-19) as a new challenge of sustainable development was investigated, using two machine learning techniques. For this purpose, we evaluated several notable climate parameters and an urban parameter, in order to find a relationship between them and the confirmed cases of COVID-19. For this, two artificial intelligence techniques, including ANN based on the PSO algorithm and DE algorithm, were used to predict the confirmed cases of COVID-19 with highly acceptable degrees of accuracy and robustness, in order to prioritize and reduce input parameters. The obtained results indicated that both developed models by PSO and DE algorithms were able to select the urban parameter and relative humidity from other effective parameters. In addition, although the two developed models had the high capability in predictive process with best costs equal to 0.0013 and 0.0014 for the PSO and DE algorithms, respectively, the developed model by PSO algorithm was a more efficient approach, compared to the other predictive method. Finally, the results were tested by a MLR and PSLR, which described the correlation between the urban parameter and relative humidity and the confirmed cases of COVID-19, with R^2^ equal to 0.76 for both regression models. For future studies, it is recommended to focus on other algorithms, other parameters for proper feature selections, and other types of climate.

## Figures and Tables

**Table 1:** The selected case studies.

Case Study	Population [92]	Density, Population/km^2^ [93]	Total Confirmed Cases Until 24th March [94]
Lombardy (Milan)	10,060,574	422	30,703
Veneto (Venice)	4,905,854	272	5948
Piedmont (Turin)	4,356,406	172	5524
Emilia-Romagna (Bolonia)	4,459,477	199	9254

**Table 2:** The control parameters of the developed model for ANN-PSO.

Control Parameters	Values
Number of hidden layers	10
Swarm size	15
Individual learning factor (C1)	1.49
Social learning factor (C2)	1.49
Maximum number of iterations	30

**Table A1:**
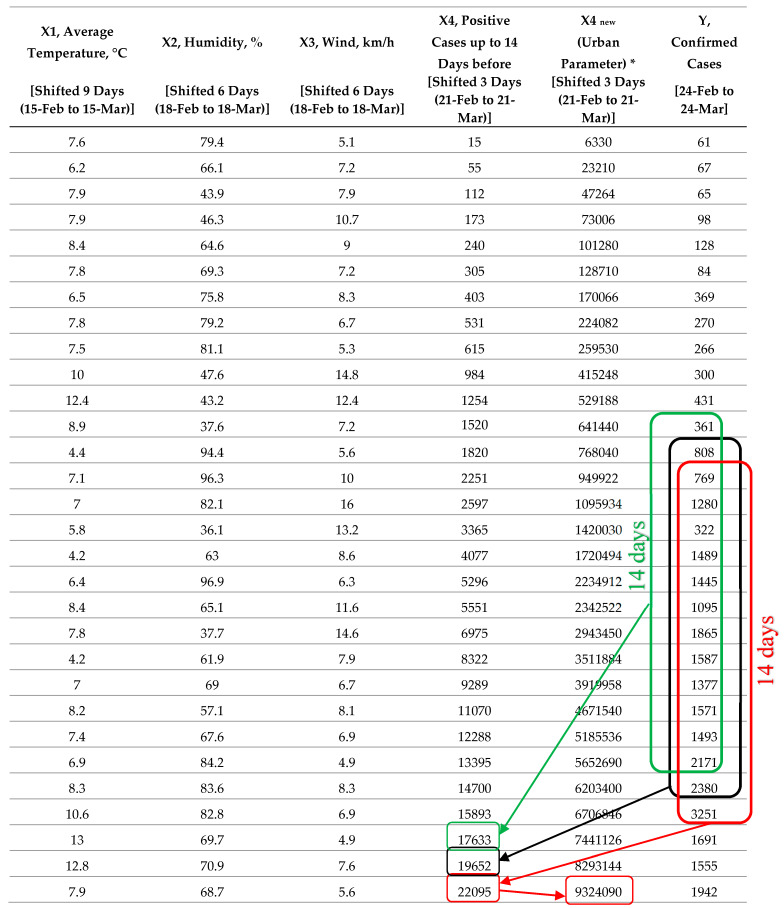
The dataset of Lombardy (Milan) according to results of multivariate linear regression (MLR), [100,101].

* Urban parameter is population density * positive cases up to 14 days before. $y=-472.213-72.474x_{1}+10.664x_{2}+73.148x_{3}+0.119x_{4}$, R^2^ = 0.79.

**Figure A1:**
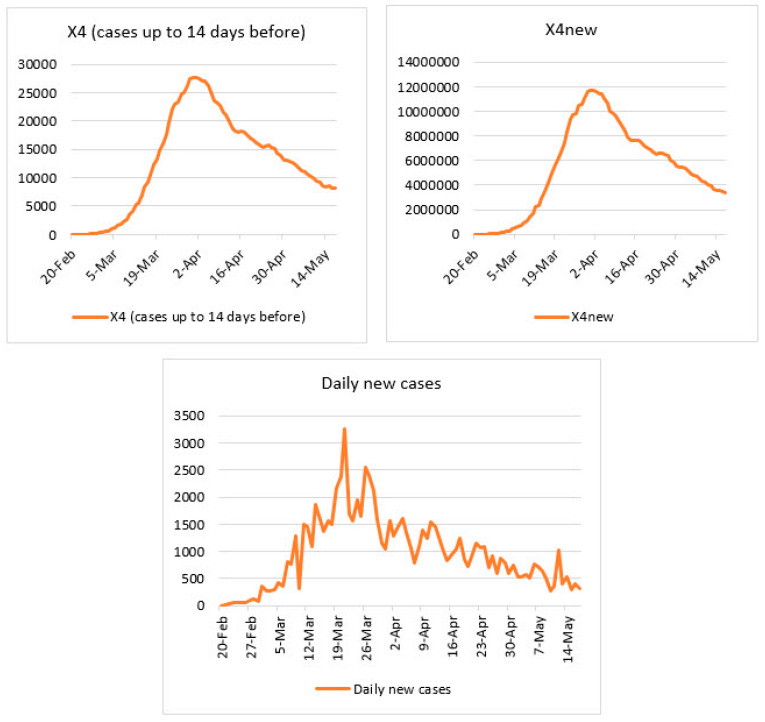
Confirmed cases of COVID-19 based on the variables of Table A5.

## Appendix A

**Table A2:** The dataset of Piedmont (Turin), according to results of MLR, [100,101].

X1, Average Temperature, °C	X2, Humidity, %	X3, Wind, km/h	X4, Positive Cases up to 14 Days before	X4 _new_ (Urban Parameter) *	Y, Confirmed Cases
[Shifted 6 Days (24-Feb to 18-Mar)]	[Shifted 9 Days (21-Feb to 15-Mar)]	[Shifted 9 Days (21-Feb to 15-Mar)]	[Shifted 2 Days (28-Feb to 22-Mar)]	[Shifted 2 Days (28-Feb to 22-Mar]	[1-Mar to 24-Mar]
5.2	72.7	2.9	10	1720	38
10.2	63.2	3.2	10	1720	2
7.0	73.4	3.0	48	8256	5
2.9	78.3	4.3	50	8600	26
2.7	78.7	5.1	55	9460	26
3.5	64.7	14.3	81	13932	35
4.6	39.6	13.7	107	18404	64
3.1	58.7	6.3	142	24424	153
3.8	49	3.8	206	35432	-
4.1	72.4	4.3	359	61748	103
4.8	80.6	4.3	359	61748	48
2.6	78.1	7.5	462	79464	79
2.5	51.9	4.9	510	87720	260
4.9	58.1	6.3	588	101136	33
5.8	90.0	5.4	839	144308	238
4.1	72.8	3.7	872	149984	405
7.2	48.9	5.2	1072	184384	381
9.8	62.6	4.3	1475	253700	444
11.0	70.0	5.0	1851	318372	591
9.4	55.0	4.5	2269	390268	529
6.6	74.7	3.6	2834	487448	291
7.1	83.4	3.0	3328	572416	668
5.8	84.4	6.7	3555	611460	441
7.5	84.2	3.8	4070	700040	654

* Urban parameter is population density * positive cases up to 14 days before. $y=288.108+9.740x_{1}-3.219x_{2}-8.188x_{3}+0.151x_{4}$, R^2^ = 0.79.

**Table A3:** The dataset of Veneto (Venice) according to results of MLR, [100,101].

X1, Average Temperature, °C	X2, Humidity, %	X3, Wind, km/h	X4, Positive Cases up to 14 Days before	X4 _new_ (Urban Parameter) *	Y, Confirmed Cases
[Shifted 5 Days (20-Feb to 19-Mar)]	[Shifted 8 Days (17-Feb to 16-Mar)]	[Shifted 6 Days (19-Feb to 18-Mar)]	[Shifted 4 Days (21-Feb to 20-Mar)]	[Shifted 4 Days (21-Feb to 20-Mar)]	[25-Feb to 24-Mar]
7.5	92.2	5.6	2	544	11
7.2	89.9	6.3	18	4896	28
7.8	86.7	10.1	25	6800	40
7.4	74.3	7.9	32	8704	40
8.9	71.3	6.3	43	11696	40
9.3	68.2	7.9	71	19312	72
9.1	87.6	6	111	30192	10
8.2	86.4	10	151	41072	34
9.1	92.7	14.6	191	51952	53
7.3	90	10.7	263	71536	47
8.6	62.6	9	273	74256	81
7.1	52.5	9.7	307	83504	55
10	64.2	11.6	360	97920	127
9.1	79.8	14.8	407	110704	74
7.2	94.8	10.7	486	132192	112
7.5	89.8	5.6	525	142800	167
8.9	76.2	16.7	645	175440	361
9.4	72.8	5.3	712	193664	211
8.6	80.7	11.4	813	221136	342
9.1	82.5	7.9	952	258944	235
9.1	68	6.5	1273	346256	301
9.2	68.2	6.7	1444	392768	231
11.2	74.2	7.9	1746	474912	510
11.5	82.3	6.7	1909	519248	270
9	84.9	14.1	2200	598400	547
8.2	89.8	17.6	2397	651984	586
9	73.8	9.3	2854	776288	505
11.1	64.3	7.2	3077	836944	383
14.2	52.6	7.2	3543	963696	443

* Urban parameter is population density * positive cases up to 14 days before. $y=1.477-10.341x_{1}+0.457x_{2}+11.467x_{3}+0.165x_{4}$, R^2^ = 0.82.

**Table A4:** The dataset of Emilia-Romagna (Bologna) according to results of MLR, [100,101].

X1, Average Temperature, °C	X2, Humidity, %	X3, Wind, km/h	X4, Positive Cases up to 14 Days before	X4 _new_ (Urban Parameter) *	Y, Confirmed Cases
[Shifted 8 days (17-Feb to 16-Mar)]	[Shifted 6 days (19-Feb to 18-Mar)]	[Shifted 8 days (17-Feb to 16-Mar)]	[Shifted 3 days (22-Feb to1-Mar)]	[Shifted 3 days (22-Feb to1-Mar)]	[25-Feb to 24-Mar]
8.8	83.3	5.6	2	398	8
11.5	72.9	6.5	9	1791	21
10.2	74	6.3	18	3582	50
8.0	74.2	7.6	26	5174	48
9	73.2	4.3	47	9353	72
7.2	77.6	5.3	97	19303	68
8.8	84.3	7.4	145	28855	50
10	69.6	4.9	217	43183	85
9.8	33.6	5.8	285	56715	124
11.2	37.8	10.2	335	66665	154
8.6	36.1	20.8	420	83580	172
10.5	66.2	19.1	544	108256	140
8.6	90.5	7.2	698	138902	170
10	82.8	12	870	173130	206
6.2	84.9	7.9	1008	200592	147
10.3	69	14.1	1171	233029	206
7.5	82.5	9.7	1368	272232	208
7.5	81.3	8.1	1507	299893	316
8.5	74.3	13.9	1692	336708	381
8.7	59.9	10.6	1850	368150	449
8.8	70.6	6.7	2118	421482	429
8.6	62.9	7.6	2427	482973	409
8.1	61.7	7.4	2808	558792	594
9.1	74	6.3	3187	634213	689
11.5	79.6	8.1	3511	698689	754
13	71.1	5.1	3981	792219	737
13.2	56.4	9	4516	898684	850
9.2	54.4	8.3	5098	1014502	980
7.6	59	6.7	5695	1133305	719

* Urban parameter is population density * positive cases up to 14 days before. $y=-64.970+14.653x_{1}-0.327x_{2}+1.616x_{3}+0.162x_{4}$, R^2^ = 0.94.

## Appendix B

The validity of using the sum of 14 days for confirmed cases for the updated data of Lombardy (Milan) until 17 April. The dataset and the graph as bellow:

**Table A5:** The updated dataset of Lombardy (Milan).

Date	Daily New Cases	X4 Positive Cases up to 14 Days before [Shifted 3 Days]	X4new [Population Density *X4]	Date	Daily New Cases	X4 Positive Cases up to 14 Days before [Shifted 3 Days]	X4new [Population Density *X4]
20-Feb	0	0	0	04-Apr	1598	27060	11419320
21-Feb	15	0	0	05-Apr	1337	26181	11048382
22-Feb	40	0	0	06-Apr	1079	25256	10658032
23-Feb	57	0	0	07-Apr	791	23603	9960466
24-Feb	61	15	6330	08-Apr	1089	23249	9811078
25-Feb	67	55	23210	09-Apr	1388	22773	9610206
26-Feb	65	112	47264	10-Apr	1246	21622	9124484
27-Feb	98	173	73006	11-Apr	1544	21068	8890696
28-Feb	128	240	101280	12-Apr	1460	19913	8403286
29-Feb	84	305	128710	13-Apr	1262	18750	7912500
01-Mar	369	403	170066	14-Apr	1012	18177	7670694
02-Mar	270	531	224082	15-Apr	827	18045	7614990
03-Mar	266	615	259530	16-Apr	941	18153	7660566
04-Mar	300	984	415248	17-Apr	1041	18118	7645796
05-Mar	431	1254	529188	18-Apr	1246	17380	7334360
06-Mar	361	1520	641440	19-Apr	855	17029	7186238
07-Mar	808	1820	768040	20-Apr	735	16615	7011530
08-Mar	769	2251	949922	21-Apr	960	16263	6862986
09-Mar	1280	2597	1095934	22-Apr	1161	15781	6659582
10-Mar	322	3365	1420030	23-Apr	1073	15437	6514414
11-Mar	1489	4077	1720494	24-Apr	1091	15606	6585732
12-Mar	1445	5296	2234912	25-Apr	713	15678	6616116
13-Mar	1095	5551	2342522	26-Apr	920	15363	6483186
14-Mar	1865	6975	2943450	27-Apr	590	15208	6417776
15-Mar	1587	8322	3511884	28-Apr	869	14377	6067094
16-Mar	1377	9289	3919958	29-Apr	786	13837	5839214
17-Mar	1571	11070	4671540	30-Apr	598	13165	5555630
18-Mar	1493	12288	5185536	01-May	737	13022	5495284
19-Mar	2171	13395	5652690	02-May	533	12981	5477982
20-Mar	2380	14700	6203400	03-May	526	12638	5333236
21-Mar	3251	15893	6706846	04-May	577	12334	5204948
22-Mar	1691	17633	7441126	05-May	500	11621	4904062
23-Mar	1555	19652	8293144	06-May	764	11292	4765224
24-Mar	1942	22095	9324090	07-May	720	11134	4698548
25-Mar	1643	23017	9713174	08-May	634	10674	4504428
26-Mar	2543	23292	9829224	09-May	502	10277	4336894
27-Mar	2409	24912	10512864	10-May	282	9924	4187928
28-Mar	2117	25066	10577852	11-May	364	9467	3995074
29-Mar	1592	26164	11041208	12-May	1033	9256	3906032
30-Mar	1154	27478	11595716	13-May	394	8618	3636796
31-Mar	1047	27730	11702060	14-May	522	8392	3541424
01-Apr	1565	27735	11704170	15-May	299	8556	3610632
02-Apr	1292	27512	11610064	16-May	399	8164	3445208
03-Apr	1455	26988	11388936	17-May	326	8088	3413136

As, the graphs show, neither X4 nor X4new reached a plateau. Thus, the mentioned method for X4, using the shifted sum of 14 days previously that is in line with the COVID-19 incubation period, might be more exact than using daily confirmed cases.

## References

[1] Kates R.W., Parris T.M., Leiserowitz A.A. (2005). What is sustainable development? Goals, indicators, values, and practice. Environment.

[2] Blewitt J. (2012). Understanding Sustainable Development.

[3] Carbone M., Garofalo G., Nigro G., Piro P. (2014). A conceptual model for predicting hydraulic behaviour of a green roof. Procedia Eng..

[4] Sustainable Development Goals. https://sustainabledevelopment.un.org/?menu=1300.

[5] Chen D., Schudeleit T., Posselt G., Thiede S. (2013). A state-of-the-art review and evaluation of tools for factory sustainability assessment. Procedia Cirp.

[6] Jayal A.D., Badurdeen F., Dillon O.W., Jawahir I.S. (2010). Sustainable manufacturing: Modeling and optimization challenges at the product, process and system levels. Cirp J. Manuf. Sci. Technol..

[7] Maiolo M., Carini M., Capano G., Piro P. (2017). Synthetic sustainability index (SSI) based on life cycle assessment approach of low impact development in the Mediterranean area. Cogent Eng..

[8] Piro P., Turco M., Palermo S.A., Principato F., Brunetti G. (2019). A Comprehensive Approach to Stormwater Management Problems in the Next Generation Drainage Networks. The Internet of Things for Smart Urban Ecosystems.

[9] Pirouz B., Arcuri N., Pirouz B., Palermo S.A., Turco M., Maiolo M. (2020). Development of an assessment method for evaluation of sustainable factories. Sustainability.

[10] Piro P., Carbone M., Garofalo G., Sansalone J. (2007). CSO treatment strategy based on constituent index relationships in a highly urbanised catchment. Water Sci. Technol..

[11] Palermo S.A., Talarico V.C., Pirouz B., Sergeyev Y., Kvasov D. (2020). Optimizing Rainwater Harvesting Systems for Non-potable Water Uses and Surface Runoff Mitigation. Numerical Computations: Theory and Algorithms. NUMTA 2019.

[12] Pirouz B., Maiolo M. (2018). The Role of Power Consumption and Type of Air Conditioner in Direct and Indirect Water Consumption. J. Sustain. Dev. Energy Water Environ. Syst..

[13] Maiolo M., Pirouz B., Bruno R., Palermo S.A., Arcuri N., Piro P. (2020). The Role of the Extensive Green Roofs on Decreasing Building Energy Consumption in the Mediterranean Climate. Sustainability.

[14] Pirouz B., Arcuri N., Maiolo M., Talarico V.C., Piro P. (2020). A new multi-objective dynamic model to close the gaps in sustainable development of industrial sector. IOP Conference Series: Earth and Environmental Science.

[15] Pirouz B., Golmohammadi A., Saeidpour Masouleh H., Violini G., Pirouz B. (2020). Relationship between average daily temperature and average cumulative daily rate of confirmed cases of COVID-19. medRixv.

[16] Pirouz B., Haghshenas S.S., Pirouz B., Haghshenas S.S., Piro P. (2020). Development of an Assessment Method for Investigating the Impact of Climate and Urban Parameters in Confirmed Cases of COVID-19: A New Challenge in Sustainable Development. Int. J. Environ. Res. Public Health.

[17] Palermo S.A., Zischg J., Sitzenfrei R., Rauch W., Piro P., Mannina G. (2019). Parameter Sensitivity of a Microscale Hydrodynamic Model. New Trends in Urban Drainage Modelling. UDM 2018.

[18] Giordano A., Spezzano G., Vinci A., Garofalo G., Piro P., Fortino G., Di Fatta G., Li W., Ochoa S., Cuzzocrea A., Pathan M. (2014). A Cyber-Physical System for Distributed Real-Time Control of Urban Drainage Networks in Smart Cities. Internet and Distributed Computing Systems. IDCS 2014.

[19] Piro P., Carbone M., Morimanno F., Palermo S.A. (2019). Simple flowmeter device for LID systems: From laboratory procedure to full-scale implementation. Flow Meas. Instrum..

[20] Carbone M., Turco M., Brunetti G., Piro P. (2015). A Cumulative Rainfall Function for Subhourly Design Storm in Mediterranean Urban Areas. Adv. Meteorol..

[21] World Health Organization (WHO) Coronavirus Disease (COVID-2019) Situation Reports. https://www.who.int/emergencies/diseases/novel-coronavirus-2019/situation-reports/.

[22] Cascella M., Rajnik M., Cuomo A., Dulebohn S.C., Di Napoli R. (2020). Features, Evaluation and Treatment Coronavirus (COVID-19).

[23] Shen M., Peng Z., Guo Y., Xiao Y., Zhang L. (2020). Lockdown may partially halt the spread of 2019 novel coronavirus in Hubei province, China. medRxiv.

[24] Phan L.T., Nguyen T.V., Luong Q.C., Nguyen T.V., Nguyen H.T., Le H.Q., Nguyen T.T., Cao T.M., Pham Q.D. (2020). Importation and human-to-human transmission of a novel coronavirus in Vietnam. N. Engl. J. Med..

[25] Gilbert M., Pullano G., Pinotti F., Valdano E., Poletto C., Boëlle P.Y., D’Ortenzio E., Yazdanpanah Y., Eholie S.P., Gutierrez B. (2020). Preparedness and vulnerability of African countries against importations of COVID-19: A modelling study. Lancet.

[26] Yu H., Sun X., Solvang W.D., Zhao X. (2020). Reverse Logistics Network Design for Effective Management of Medical Waste in Epidemic Outbreaks: Insights from the Coronavirus Disease 2019 (COVID-19) Outbreak in Wuhan (China). Int. J. Environ. Res. Public Health.

[27] Chen Y.C., Lu P.E., Chang C.S. (2020). A Time-dependent SIR model for COVID-19. arXiv.

[28] Pirouz B., Shaffiee Haghshenas S., Shaffiee Haghshenas S., Piro P. (2020). Investigating a Serious Challenge in the Sustainable Development Process: Analysis of Confirmed cases of COVID-19 (New Type of Coronavirus) Through a Binary Classification Using Artificial Intelligence and Regression Analysis. Sustainability.

[29] Hu Z., Ge Q., Jin L., Xiong M. (2020). Artificial intelligence forecasting of covid-19 in china. arXiv.

[30] Kampf G., Todt D., Pfaender S., Steinmann E. (2020). Persistence of coronaviruses on inanimate surfaces and its inactivation with biocidal agents. J. Hosp. Infect..

[31] Grant W.B., Giovannucci E. (2009). The possible roles of solar ultraviolet-B radiation and vitamin D in reducing case-fatality rates from the 1918–1919 influenza pandemic in the United States. Derm. Endocrinol..

[32] Chan K.H., Peiris J.S., Lam S.Y., Poon L.L., Yuen K.Y., Seto W.H. (2011). The effects of temperature and relative humidity on the viability of the SARS coronavirus. Adv. Virol..

[33] Coronavirus Incubation Period. https://www.worldometers.info/coronavirus/coronavirus-incubation-period/#24.

[34] Coronavirus Testing: How it Is Done, When you Should Get One and How Long Results Take. https://www.liverpoolecho.co.uk/news/uk-world-news/coronavirus-testing-how-done-you-17912266.

[35] Li Q., Guan X., Wu P., Wang X., Zhou L., Tong Y., Ren R., Leung K.S.M., Lau E.H.Y., Xing X. (2020). Early Transmission Dynamics in Wuhan, China, of Novel Coronavirus–Infected Pneumonia. N. Engl. J. Med..

[36] Coronavirus Testing: Information on COVID-19 Tests According to State Health Departments. https://www.nbcnews.com/health/health-news/coronavirus-testing-information-covid-19-tests-according-state-health-departments-n1158041.

[37] In Italia ieri Record Mondiale di Decessi Rebus Lombardia. https://ilmanifesto.it/in-italia-ieri-record-mondiale-di-decessi-rebus-lombardia/.

[38] Coronavirus Cases. https://www.worldometers.info/coronavirus/.

[39] Mikaeil R., Haghshenas S.S., Hoseinie S.H. (2018). Rock penetrability classification using artificial bee colony (ABC) algorithm and self-organizing map. Geotech. Geol. Eng..

[40] Naderpour H., Mirrashid M. (2019). Moment capacity estimation of spirally reinforced concrete columns using ANFIS. Complex Intell. Syst..

[41] Naderpour H., Mirrashid M. (2019). Shear failure capacity prediction of concrete beam–column joints in terms of ANFIS and GMDH. Pract. Period. Struct. Des. Constr..

[42] Kayabekir A.E., Toklu Y.C., Bekdaş G., Nigdeli S.M., Yücel M., Geem Z.W. (2020). A Novel Hybrid Harmony Search Approach for the Analysis of Plane Stress Systems via Total Potential Optimization. Appl. Sci..

[43] Na K.S., Cho S.E., Geem Z.W., Kim Y.K. (2020). Predicting future onset of depression among community dwelling adults in the Republic of Korea using a machine learning algorithm. Neurosci. Lett..

[44] Kandiri A., Golafshani E.M., Behnood A. (2020). Estimation of the compressive strength of concretes containing ground granulated blast furnace slag using hybridized multi-objective ANN and salp swarm algorithm. Constr. Build. Mater..

[45] Geem Z.W. (2011). Transport energy demand modeling of South Korea using artificial neural network. Energy Policy.

[46] Rad M.Y., Haghshenas S.S., Kanafi P.R., Haghshenas S.S. Analysis of Protection of Body Slope in the Rockfill Reservoir Dams on the Basis of Fuzzy Logic. Proceedings of the 4th International Joint Conference on Computational Intelligence.

[47] Rad M.Y., Haghshenas S.S., Haghshenas S.S. Mechanostratigraphy of cretaceous rocks by fuzzy logic in East Arak, Iran. Proceedings of the 4th International Workshop on Computer Science and Engineering-Summer.

[48] Mikaeil R., Haghshenas S.S., Haghshenas S.S., Ataei M. (2018). Performance prediction of circular saw machine using imperialist competitive algorithm and fuzzy clustering technique. Neural Comput. Appl..

[49] Dormishi A., Ataei M., Mikaeil R., Khalokakaei R., Haghshenas S.S. (2019). Evaluation of gang saws’ performance in the carbonate rock cutting process using feasibility of intelligent approaches. Eng. Sci. Technol. Int. J..

[50] Mikaeil R., Beigmohammadi M., Bakhtavar E., Haghshenas S.S. (2019). Assessment of risks of tunneling project in Iran using artificial bee colony algorithm. SN Appl. Sci..

[51] Behnood A., Golafshani E.M. (2020). Machine learning study of the mechanical properties of concretes containing waste foundry sand. Constr. Build. Mater..

[52] Cabaneros S.M.S., Calautit J.K., Hughes B.R. (2019). A review of artificial neural network models for ambient air pollution prediction. Environ. Model. Softw..

[53] Maleki H., Sorooshian A., Goudarzi G., Baboli Z., Birgani Y.T., Rahmati M. (2019). Air pollution prediction by using an artificial neural network model. Clean Technol. Environ. Policy.

[54] Araujo L.N., Belotti J.T., Alves T.A., de Souza Tadano Y., Siqueira H. (2020). Ensemble method based on Artificial Neural Networks to estimate air pollution health risks. Environ. Model. Softw..

[55] Mahdevari S., Shahriar K., Sharifzadeh M., Tannant D.D. (2017). Stability prediction of gate roadways in longwall mining using artificial neural networks. Neural Comput. Appl..

[56] Naderpour H., Poursaeidi O., Ahmadi M. (2018). Shear resistance prediction of concrete beams reinforced by FRP bars using artificial neural networks. Measurement.

[57] Mohammadi J., Ataei M., Kakaei R.K., Mikaeil R., Haghshenas S.S. (2018). Prediction of the production rate of chain saw machine using the multilayer perceptron (MLP) neural network. Civ. Eng. J..

[58] Mikaeil R., Haghshenas S.S., Ozcelik Y., Gharehgheshlagh H.H. (2018). Performance evaluation of adaptive neuro-fuzzy inference system and group method of data handling-type neural network for estimating wear rate of diamond wire saw. Geotech. Geol. Eng..

[59] Genc O., Kisi O., Ardiclioglu M. (2019). Modeling velocity distributions in small streams using different neuro-fuzzy and neural computing techniques. J. Water Clim. Chang..

[60] Faris H., Aljarah I., Mirjalili S. (2018). Improved monarch butterfly optimization for unconstrained global search and neural network training. Appl. Intell..

[61] Noori A.M., Mikaeil R., Mokhtarian M., Haghshenas S.S., Foroughi M. (2020). Feasibility of intelligent models for prediction of utilization factor of TBM. Geotech. Geol. Eng..

[62] Petrudi S.H.J., Pirouz M., Pirouz B. Application of fuzzy logic for performance evaluation of academic students. Proceedings of the 13th Iranian Conference on Fuzzy Systems (IFSC).

[63] Karahan H., Gurarslan G., Geem Z.W. (2013). Parameter estimation of the nonlinear Muskingum flood-routing model using a hybrid harmony search algorithm. J. Hydrol. Eng..

[64] Haghshenas S.S., Haghshenas S.S., Mikaeil R., Sirati Moghadam P., Haghshenas A.S. (2017). A new model for evaluating the geological risk based on geomechanical properties-case study: The second part of emamzade hashem tunnel. Electron. J. Geotech. Eng..

[65] Salemi A., Mikaeil R., Haghshenas S.S. (2018). Integration of finite difference method and genetic algorithm to seismic analysis of circular shallow tunnels (Case study: Tabriz urban railway tunnels). KSCE J. Civ. Eng..

[66] Haghshenas S.S., Faradonbeh R.S., Mikaeil R., Haghshenas S.S., Taheri A., Saghatforoush A., Dormishi A. (2019). A new conventional criterion for the performance evaluation of gang saw machines. Measurement.

[67] Roohollah Shirani F., Haghshenas S.S., Taheri A., Mikaeil R. (2019). Application of self-organizing map and fuzzy c-mean techniques for rockburst clustering in deep underground projects. Neural Comput. Appl..

[68] Mikaeil R., Bakhshinezhad H., Haghshenas S.S., Ataei M. (2019). Stability analysis of tunnel support systems using numerical and intelligent simulations (case study: Kouhin Tunnel of Qazvin-Rasht Railway). Rud. Geološko Naft. Zb..

[69] Hosseini S.M., Ataei M., Khalokakaei R., Mikaeil R., Haghshenas S.S. (2020). Study of the effect of the cooling and lubricant fluid on the cutting performance of dimension stone through artificial intelligence models. Eng. Sci. Technol. Int. J..

[70] Tikhamarine Y., Souag-Gamane D., Ahmed A.N., Kisi O., El-Shafie A. (2020). Improving artificial intelligence models accuracy for monthly streamflow forecasting using grey Wolf optimization (GWO) algorithm. J. Hydrol..

[71] Pirouz B., Palermo S.A., Turco M., Piro P., Sergeyev Y., Kvasov D. (2020). New Mathematical Optimization Approaches for LID Systems. Numerical Computations: Theory and Algorithms. NUMTA 2019.

[72] Ho C.H., Chang P.T., Hung K.C., Lin K.P. (2019). Developing intuitionistic fuzzy seasonality regression with particle swarm optimization for air pollution forecasting. Ind. Manag. Data Syst..

[73] Lee C.Y., Lee Z.J., Huang J.Q., Ye F.L., Ning Z.Y., Yang C.F. (2019). Urban Air Quality Analysis and Forecast Based on Intelligent Algorithm with Parameter Optimization and Decision Rules. Appl. Sci..

[74] Shamshirband S., Hadipoor M., Baghban A., Mosavi A., Bukor J., Várkonyi-Kóczy A.R. (2019). Developing an ANFIS-PSO model to predict mercury emissions in combustion flue gases. Mathematics.

[75] Wang J., Du P., Hao Y., Ma X., Niu T., Yang W. (2020). An innovative hybrid model based on outlier detection and correction algorithm and heuristic intelligent optimization algorithm for daily air quality index forecasting. J. Environ. Manag..

[76] Zeinalnezhad M., Chofreh A.G., Goni F.A., Klemeš J. (2020). Air pollution prediction using semi-experimental regression model and Adaptive Neuro-Fuzzy Inference System. J. Clean. Prod.

[77] Kennedy J., Eberhart R. Particle swarm optimization. Proceedings of the ICNN’95-International Conference on Neural Networks.

[78] Shi Y. Particle swarm optimization: Developments, applications and resources. Proceedings of the 2001 Congress on Evolutionary Computation.

[79] Poli R., Kennedy J., Blackwell T. (2007). Particle swarm optimization. Swarm Intell..

[80] Mikaeil R., Haghshenas S.S., Sedaghati Z. (2019). Geotechnical risk evaluation of tunneling projects using optimization techniques (case study: The second part of Emamzade Hashem tunnel). Nat. Hazards.

[81] Karaboga D., Akay B. (2019). A comparative study of artificial bee colony algorithm. Appl. Math. Comput..

[82] Tharwat A., Elhoseny M., Hassanien A.E., Gabel T., Kumar A. (2019). Intelligent Bézier curve-based path planning model using Chaotic Particle Swarm Optimization algorithm. Clust. Comput..

[83] Aryafar A., Mikaeil R., Doulati Ardejani F., Shaffiee Haghshenas S., Jafarpour A. (2019). Application of non-linear regression and soft computing techniques for modeling process of pollutant adsorption from industrial wastewaters. J. Min. Environ..

[84] Storn R., Price K. (1995). Differential Evolution—A Simple and Efficient Adaptive Scheme for Global Optimization over Continuous Spaces.

[85] Storn R., Price K. Minimizing the real functions of the ICEC’96 contest by differential evolution, In Evolutionary Computation. Proceedings of the IEEE International Conference on Evolutionary Computation.

[86] Storn R., Price K. (1997). Differential evolution–a simple and efficient heuristic for global optimization over continuous spaces. J. Glob. Optim..

[87] Aryafar A., Mikaeil R., Haghshenas S.S., Haghshenas S.S. (2018). Application of meta-heuristic algorithms to optimal clustering of sawing machine vibration. Measurement.

[88] Dehghani H., Shafaghi M. (2017). Prediction of blast-induced flyrock using differential evolution algorithm. Eng. Comput..

[89] Yang J., Li W.T., Shi X.W., Xin L., Yu J.F. (2013). A hybrid ABC-DE algorithm and its application for time-modulated arrays pattern synthesis. IEEE Trans. Antennas Propag..

[90] Wang L., He J., Wu D., Zeng Y.R. (2012). A novel differential evolution algorithm for joint replenishment problem under interdependence and its application. Int. J. Prod. Econ..

[91] Dormishi A.R., Ataei M., Khaloo Kakaie R., Mikaeil R., Shaffiee Haghshenas S. (2019). Performance evaluation of gang saw using hybrid ANFIS-DE and hybrid ANFIS-PSO algorithms. J. Min. Environ..

[92] AdminStat Maps, Analysis and Statistics about the Resident Population. https://ugeo.urbistat.com/AdminStat/en/it/demografia/dati-sintesi/milano/15/3.

[93] Regions of Italy. https://en.wikipedia.org/wiki/Regions_of_Italy.

[94] 2020 Coronavirus Pandemic in Italy. https://en.wikipedia.org/wiki/2020_coronavirus_pandemic_in_Italy#cite_note-139.

[95] Lombardy. https://commons.wikimedia.org/wiki/File:Map_of_region_of_Lombardy,_Italy,_with_provinces-it.svg.

[96] Piedmont. https://commons.wikimedia.org/wiki/File:Map_of_region_of_Piedmont,_Italy,_with_provinces-en.svg.

[97] Emilia-Romagna. https://en.wikipedia.org/wiki/Emilia-Romagna#/media/File:Map_of_region_of_Emilia-Romagna,_Italy,_with_provinces-it.svg.

[98] Veneto. https://en.wikipedia.org/wiki/File:Map_of_region_of_Veneto,_Italy,_with_provinces-en.svg.

[99] Nelson M.M., Illingworth W.T. (1991). A Practical Guide to Neural Nets.

[100] Meteorological Conditions in the World. https://www.ogimet.com/ranking.phtml.en.

[101] Weather Forecast. https://yandex.com/weather/bologna/month/february.

